# Abstracts of papers presented at the 29^th^ Genetic Society's Mammalian Genetics and Development Workshop held at the UCL Great Ormond Street Institute of Child Health, University College London on Thursday 29^th^ November 2018

**DOI:** 10.1017/S0016672319000016

**Published:** 2019-03-11

**Affiliations:** 1 UCL Great Ormond Street Institute of Child Health, 30 Guilford Street, London, WC1N 1EH; 2 KCL Craniofacial Development and Stem Cell Biology, Floor 27 Tower Wing, Guy's Campus SE1 9RT

Trisomy of human chromosome 21, which causes Down syndrome (DS), is the most common genetic cause of dementia. By the age of 40, virtually all people with DS have amyloid plaques and neurofibrillary tangles, the main pathological hallmarks of Alzheimer disease (AD). The main component of amyloid plaques, amyloid beta, is produced through sequential cleavage of Amyloid Precursor Protein (APP). The *APP* gene is present on chromosome 21, but other genes are likely to also have a role in AD in DS (AD-DS). Our aim is to study the effect of triplication of chromosome 21 genes other than *APP* on APP processing. We use Mouse Embryonic Fibroblasts (MEFs) derived from a DS mouse model (Dp1Tyb) that carries a segmental duplication of 23Mb of mouse chromosome 16, homologous to chromosome 21. We will focus on the expression and half-life of APP-derived fragments and on alteration in the structure of endosomes, a main site of APP processing. The ultimate aim of our research is to identify the genes responsible for altered APP processing in AD-DS. To do this we plan to use mouse models that have segmental duplications of progressively smaller regions of the mouse chromosomes homologous of human chromosome 21.

