# Supplementary material for: Abstracts of papers presented at the 29th Genetic Society's Mammalian Genetics and Development Workshop held at the UCL Great Ormond Street Institute of Child Health, University College London on Thursday 29th November 2018
Source: Genet Res (Camb). 2019 Mar 11;101:e4. doi: 10.1017/S0016672319000016 (PMC7045033; doi:10.1017/S0016672319000016)
Supplement: Related texts

# Abstract: Cohesin is continuously required to sustain neuronal gene expression

**Authors:** Lesly Calderon, Felix D Weiss, Thomas Carroll, Elaine E. Irvine, Gopuraja Dharmalingam, Kyoko Tossell, Vincenzo De Paola, Chad Whilding, Mark A. Ungless, Dominic J. Withers, Amanda G Fisher, Matthias Merkenschlager

**Affiliations:** 1 MRC London Institute of Medical Sciences, Institute of Clinical Sciences, Faculty of Medicine, Imperial College London, Du Cane Road, London W12 0NN, UK; 2 Institute of Clinical Sciences, Faculty of Medicine, Imperial College London, Du Cane Road, London W12 0NN, UK

Cornelia de Lange Syndrome (CdLS) is a developmental disorder caused by mutations that compromise the function of cohesin, a major regulator of 3-D genome organisation. A universal yet unexplained feature of CdLS is cognitive impairment. In particular, it is not known whether neuronal dysfunction arises primarily from developmental defects in neuronal progenitors, or from an ongoing requirement for cohesin in post-mitotic neurons. To address these questions, we investigated the impact on neuronal gene expression of depleting cohesin at distinct points in development - in the germline, in immature post-mitotic neurons, or in mature neurons. Unexpectedly, neuronal genes with key roles in synaptic transmission, connectivity, neuronal development and signaling were selectively downregulated, regardless of whether cohesin function was compromised throughout development, during the differentiation of immature post-mitotic neurons, or in mature neurons for just 24 hours. Our findings establish that cohesin is required to sustain the expression of functionally important neuronal genes, and provide a tractable approach to neuronal dysfunction in CdLS.

# Abstract: Is apical constriction synchronised with cell cycle progression in mammalian neurulation?

**Authors:** Max B Butler, Nina Short, Gabriel L Galea

**Affiliations:** 1 Wellcome Biomedical Vacation Scholar, UCL GOS Institute of Child Health; 2 CHRAT vacation student, UCL GOS Institute of Child Health; 3 Developmental Biology of Birth Defects, UCL GOS Institute of Child Health

Embryonic epithelia generate mechanical forces which sculpt the embryo. During neural tube closure, actomyosin-dependant neuroepithelial apical constriction and interkinetic nuclear migration (IKNM) facilitate apposition of the posterior neuropore (PNP) neural folds. Both apical constriction and IKNM are force generating mechanisms which co-regulate epithelial apical dimensions, but how they co-exist in the neuroepithelium is unknown.

To investigate this, E9 CD1 mouse embryos were cultured in the Rho-associated kinase (ROCK) inhibitor Y-27632. Progression past S-phase was blocked with hydroxyurea. Whole-mount confocal immunofluroscopy was used to visualise cell cycle markers (G2→M: pHH3, M→G1: pRB) and cell outlines (Scrib).

ROCK inhibition widened the PNP and increased neuroepithelial apical dimensions without substantially changing mitotic index. In vehicle-treated embryos, pRB+ cells predominantly had small apical areas. ROCK inhibition (2hrs) significantly increased their apical dimensions. pHH3+ cells’ apical dimensions ranged from the smallest to the largest observed; surprisingly, this was unchanged by ROCK inhibition (2hrs). This suggests neuroepithelial cells adopt small apical dimensions independently of ROCK during M-phase, but require ROCK to maintain apical constriction when entering G1. Trapping neuroepithelial cells in S-phase for 8hrs narrowed the PNP and prevented its widening following ROCK inhibition. Thus, ROCK-dependent apical constriction is temporally coordinated with IKNM during PNP closure.

# Abstract: The role of cellular senescence and its associated secretome in paediatric Adamantinomatous Craniopharyngioma (ACP)

**Authors:** Saba Manshaei, Scott Haston, Jesus Gil, Juan Pedro Martinez-Barbera

**Affiliations:** 1 Developmental Biology and Cancer Programme, Birth Defects Research Centre, UCL Institute of Child Health, London WC1N 1EH, UK; 2 Cell Proliferation Group, MRC Clinical Sciences Centre, Imperial College London, Hammersmith Campus, London W12 0NN, UK

ACP is the most common non-neuroepithelial developmental brain-tumour. Mouse models generated in our group have demonstrated that mutations in *CTNNB1* during development can drive this tumour-formation. SOX2 + ve pituitary stem cells targeted with oncogenic beta- catenin form senescent cell clusters that can induce tumourigenesis non-cell-autonomously, through a senescent-associated-secretory-phenotype (SASP), comprised of cytokines, chemokines, growth factors, etc. To elucidate the role of senescence in ACP I am generating a mouse model (*p21^FDR/+^*) allowing detection and ablation of senescent cells *in-vivo*. Selective ablation of cluster cells at time-points during tumour formation will determine whether the cells are required for tumour initiation, or solely in tumour maintenance and growth. Additionally a *Rosa2^STOP-mBRF1/+^* mouse will attenuate activities of SASP genetically, to determine the crucial secretory drivers of tumourigenesis, as well as to determine the developmental roles of cytokines and chemokines in the pituitary. This will provide insight into senescence in ACP pathogenesis and pituitary development.

# Abstract: Epigenetic revertant mosaicism in congenital melanocytic naevi – proof of concept for allele-specific silencing

**Authors:** Melissa Riachi, Lara Al-Olabi, William Baird, Satyamaanasa Polubothu, Veronica A Kinsler

**Affiliations:** 1 Genetics and Genomic Medicine, UCL GOS Institute of Child Health, London, UK; 2 Paediatric Dermatology, Great Ormond Street Hospital for Children, London, UK

Revertant mosaicism is the process of *in vivo* correction of a mutation which results in phenotypic rescue, previously only described in autosomal recessive skin diseases with secondary somatic DNA mutations. We describe here epigenetic revertant mosaicism in three patients with congenital melanocytic naevi (CMN), a condition caused by *in utero* somatic heterozygous mutations in critical signaling pathway gene *NRAS*. Patients presented with new islands of normal skin within naevi, and biopsies were obtained from both areas. Unexpectedly, the known DNA mutation was present in all areas, as were equal numbers of naevus cells, however the expression within the phenotypically normal skin was strikingly monoallelic, and restricted to the normal allele. This was confirmed using an optimized and highly specific gene expression assay for the mutant alleles. Investigation of the underlying mechanism has excluded secondary promoter mutations in *NRAS*, and allele-specific differences in CpG methylation. This phenomenon of natural genetic therapy *in vivo* is proof of concept that therapeutic allele-specific targeting of gene expression would be sufficient to correct the clinical phenotype.

# Abstract: Refining mouse models for preclinical studies

**Authors:** Martin Fray

**Affiliations:** MRC Harwell Institute, Mary Lyon Centre, Harwell Campus, Oxfordshire, OX11 0RD, UK

MRC Harwell is involved in many national and international projects using mouse models to study the relationship between genes and disease. We have many resources available to the biomedical community which we are keen to share.

**Archiving -** We have a frozen embryo and sperm archive where we can cryopreserve your mouse strains for free. These can then be sent anywhere in the world.

**Genome Editing Mice for Medicine -** Our Genome Editing Mice for Medicine programme is an MRC programme aimed at generating scientifically important, novel mouse lines taking advantage of advances in genome editing. UK scientists are invited to nominate genetically altered mouse lines to be made to advance their own research and be of widespread use in biomedical science.

**Training -** We have a range of different training courses for laboratory and animal science. These courses not only include technical courses in transgenesis, pathology and cryopreservation but also taught courses in mouse genetics and genome editing.

**IMPC -** We are a member of the International Mouse Phenotyping Consortium (IMPC) a global programme looking to find the function of every protein-coding gene in the mouse genome, with the data freely accessible online. If you are interested in a particular gene knockout, you can nominate it through us.

# Abstract: Epithelial dynamics during development of the mammalian ear canal

**Authors:** Mona Mozaffari, Juan Fons Romero, Dean Malik, Abigail Tucker

**Affiliations:** Centre for Craniofacial and Regenerative Biology, Floor 27 Guy's Tower, King's College London, SE1 9TR

Defects in ear canal development can cause severe hearing loss, however very little is known about how the canal initiates, extends and opens. Here we have studied mammalian ear canal development and show that the canal undergoes a complex system of closure and reopening as it forms. The more superficial part of the canal formed from an open primary canal, initiating at the junction between the first and second arch, which later collapsed and then reopened. In contrast, the deeper part of the canal formed from a solid meatal plate that extended from the primary canal into the first arch and later opened. As the ear canal developed, the different parts displayed distinct patterns of proliferation and keratin expression, with collapse of the primary canal linked to loss of periderm. Final opening of the canal was triggered by terminal differentiation of the epithelium. Interestingly, the meatal plate opened asymmetrically, associated with differential proliferation, to create the thin outer surface of the ear-drum. Understanding these complex processes involved in canal development can shed light on the underlying causes of canal atresia.

# Abstract: Congenital Macular Dystrophy is caused by non-coding duplications downstream of *IRXA*locus

**Authors:** Raquel S. Silva, K. Kraft, G. Arno, V. Cipriani, V. Heinrich, N. Pontikos, B. Puech, A. Moore, V. van Heyningen, S. Mundlos, A. R. Webster

**Affiliations:** 1 UCL Institute of Ophthalmology, London, United Kingdom; 2 Moorfields Eye Hospital, London, United Kingdom; 3 Max Planck Institute for Molecular Genetics, Berlin, Germany; 4 Institute for Medical and Human Genetics, Charité Universitätsmedizin, Berlin, Germany; 5 Exploration de la Vision et Neuro-Ophtalmologie, Centre Hospitalier Universitaire, Lille, France; 6 Ophthalmology Department, UCSF School of Medicine, San Francisco, CA, United States

Autosomal dominant North Carolina Macular Dystrophy (NCMD) is believed to represent a failure of macular development with consequent central vision loss. Prior genetic linkage pinpointed the disease locus to chromosome 6q16 and 5p15. The aim was to identify causative variants and further the understanding of the disease mechanism.

CRISPR technology was used to reproduce a 50 kb non-coding tandem duplication between *IRX1* and*ADAMTS16* (chr5 in hg19 and chr13 in mm9) recurrently identified in NCMD families by whole genome sequencing. Gene expression studies were performed on developing fetal eye and limb-bud. Chromosome conformation capture (c-HI-C) and chromatin immunoprecipitation performed on the same tissues, mapped chromatin architectural folding and defined putative regulatory elements.

Expression of *IRX1* and *IRX2* was increased in the limb-bud tissue at E12·5 in the mouse model versus control. Subsequently these genes are downregulated at E14·5, suggesting temporal dysregulation of the IRXA cluster. c-Hi-C at the 5p locus showed unique chromatin interactions in developing wild-type eye versus limb-bud, suggesting distinct tissue-specific regulation.

Chromatin organization findings highlight the relevance of tissue specific DNA contacts in development and disease. Further characterization of mouse model and exploration of the nine unsolved cases may uncover additional molecular targets for NCMD.

# Abstract: Tctex1d2, an integral transporter for (Intraflagellar Transport) IFT components during sperm development

**Authors:** Mitali P Patel, Anu Sironen, Victor Hernandez-Hernandez, Gabriel Galea, Laura Dyer, Ian Simmock, Owen Arthurs, Susan Shelmerdine, Mark Turmaine, Hannah Mitchison

**Affiliations:** 1 Genetics and Genomic Medicine, University College London, UCL Great Ormond Street Institute of Child Health, London WC1N 1EH, UK; 2 Developmental Biology of Birth Defects, University College London, UCL Great Ormond Street Institute of Child Health, London WC1N 1EH, UK; 3 Mammalian Genetics Unit, MRC Harwell Institute, Harwell Campus, Oxfordshire OX11 0RD, UK; 4 Department of Radiology, Great Ormond Street Hospital for Children NHS Foundation Trust, WC1N 3JH, UK; 5 Department of Biosciences, University College London, UK

Juene syndrome is a rare autosomal recessive ciliopathy affecting about 1 in 100,000 – characterized by shortening of long bones, constriction of the thorax and polydactyly. Here we characterize a Tctex1d2 gene trap mouse model creating successful knock-down of gene expression particularly in the testes and bone. Tctex1d2 is a light chain of the ciliary intra-flagellar transport (IFT) dynein-2 motor complex, essential for motor stability and trafficking of protein cargos required for ciliogenesis. Although no gross skeletal abnormalities were evident, micro-CT scans revealed mild elevation in long bone (tibia) mass confirming a skeletal phenotype. Furthermore, a significant reduction in sperm number and motility was observed in mutant epididymis with several spermatogenesis defects including amorphous heads, short/bent flagella and swollen tail tips. During spermatid differentiation, protein transport is based on IFT and intra-manchette transport. In the mutants, Tctex1d2 protein was seen to be absent in the flagellum of developing spermatids and mature sperms. Hence, to determine the stage of spermatogenesis at which Tctex1d2 is required for cellular cargo trafficking selective IFT components were studied. We found markedly increased staining along the flagella of IFT components in the mutants, suggesting that transport of IFT proteins is disrupted. We therefore conclude that Tctex1d2 is an integral component of the sperm retrograde IFT machinery.

# Abstract: Characterisation of a SOX2-positive population in the postnatal adrenal medulla.

**Authors:** Alice Santambrogio, JP Russell, APB Brennand, EJ Lodge, C Steenblock, SR Bornstein, CL Andoniadou

**Affiliations:** 1 Centre for Craniofacial and Regenerative Biology, King's College London, London, United Kingdom University; 2 Hospital Carl Gustav Carus, Dept. of Medicine III, Technische Universität Dresden, Dresden, Germany; 3 Diabetes and Nutritional Sciences Division, King's College London, London, United Kingdom

The adrenal gland is an endocrine organ responsible for the stress response and is involved in the regulation of the immune system and metabolism. The adrenal gland is composed of an outer cortex and an inner medulla, which have distinct functions. It is a dynamic organ, capable of responding to the variable physiological demand requiring production of steroids and catecholamines. While stem and progenitor cell populations in the adrenal cortex have been identified and characterised, investigation of the adrenal medulla cell hierarchy is still ongoing.

The adrenal medulla derives from the neural crest, from which a common progenitor generates both sympathetic neurons and neuroendocrine chromaffin cells. SOX2 is a marker of multiple stem cell/progenitor lineages and is found to be upregulated in many tumours including pheochromocytomas and paragangliomas, tumours of the adrenal medulla.

Here we identify and characterise SOX2-positive cells in the murine adrenal medulla during homeostasis. SOX2-positive cells do not colocalise with other known progenitor markers in the medulla. Using genetic lineage tracing in the juvenile postnatal animal, we identify SOX2-positive cells to be an expanding population which gives rise to chromaffin cells. Taken together our data point towards a new candidate progenitor cell population of the adrenal medulla.

# Abstract: The periodic coloration in birds forms through a prepattern of somite origin

**Authors:** Nicolas Haupaix, Camille Cuurantz, Richard Bailleul, Samantha Beck, Annie Robic, Marie Manceau

**Affiliations:** 1 Center for Interdisciplinary Research in Biology, CNRS 7241, INSERM U1042, Collège de France, Paris, France; 2 GenPhySE, Toulouse University, INRA, INPT ENVT 31326, Castanet-Tolosan, France

Spots, stripes… These periodic patterns often seen in animals have been largely viewed as self-organizing. Do they also depend on preexisting positional information? In juvenile galliform birds we show that signaling from the somite sets the position of stripes in the plumage, while their width is controlled by the expression of *agouti*. These results reveal that early developmental landmarks can shape periodic patterns upstream of late local dynamics, and thus constrain their evolution.

# Abstract: Surface ectoderm biomechanics depend on Grainyhead-like protein 2 (Grhl2) to regulate neural tube closure.

**Authors:** Evanthia Nikolopoulou, Caroline Hirst, Gabriel Galea, Dale Moulding, Cristina Venturini, Abigail Marshall, Andrew Copp, Nicholas Greene

**Affiliations:** 1 Developmental Biology and Cancer Programme, UCL Great Ormond Street Institute of Child Health, University College London, 30 Guilford Street, London, WC1N 1EH; 2 UCL Infection and Immunity Division, UCL Pathogen Genomic Unit, UCL Cruciform Building, Gower Street, London WC1E 6BT

Grainyhead-like proteins (Grhls) are evolutionary conserved transcription factors essential for neural tube closure. Grhl2 knockout models display both cranial and spinal neural tube defects (NTDs), whereas the *Axial defects* (*Axd*) model, a Grhl2 overexpressing model, has spinal defects. Grhl2 is expressed in the non-neural ectoderm or surface ectoderm, a monolayer of cells covering and wrapping around the neural folds that ultimately will give rise to the epidermis. We investigated the molecular and cellular mechanisms by which either excess or insufficient Grhl2 expression can cause NTDs. We performed RNA-seq experiments on both knockout and overexpressing models (*Axd*) and identified multiple pathways regulated inversely in both models. Genes encoding proteins of the junctional apical complex were highly regulated in both datasets. We further validated a number of adherens junction molecules (such as E-cadherin) and tight junction molecules (a number of claudins) to be regulated by Grhl2 in the caudal neural tube. Consistent with the fact that these molecules are pivotal for cell-cell adhesion and connected to the cytoskeleton, we also identified abnormalities in F-actin and p-MLCII organization. In addition, absence of Grhl2 altered the nature of the surface ectoderm cells which acquired a more neuroepithelial phenotype. Finally, functional experiments showed that the surface ectoderm cells in the presence of elevated or diminished Grhl2 expression, were under abnormal tension. In conclusion, Grhl2 defines the epithelial nature of the surface ectoderm monolayer, making it a determining factor for neural tube closure.

# Abstract: Increased repair in neural crest frontal bones correlates with recruitment of sutural stem cells

**Authors:** Daniel Doro, Kshemendra Senarath-Yapa, Jiashang Lau, Annie Liu, Inuyoung Cho, Akif Reza, Agamemnom Grigoriadis, Karen Liu

**Affiliations:** Centre for Craniofacial and Regenerative Biology – King's College London, UK

The craniofacial skeleton is formed from the neural crest and mesodermal lineages, both of which contribute mesenchymal precursors during formation of frontal and parietal bones respectively. Increased healing capacity in frontal bones has been previously described. In culture, frontal bone cells are more osteogenic than parietals, as seen by formation of more mineralized nodules. Although differences in molecular signalling and osteogenic potential have been well studied, the recruitment of suture mesenchymal cells and specific stem cell subsets has not yet been investigated. Here we combine lineage tracing techniques in a calvarial wound model to assess the infiltration of previously reported stem cell populations, as well as the availability and recruitment of neural crest derivatives. 2 mm defects were performed on frontal and parietal bones of 6 weeks old mice. Gli1-Cre^ERT^; Rosa26R^mTmG^ and Axin2-Cre^ERT^; Rosa26^Tomato^ mice were induced with tamoxifen 2 days prior to osteoctomy, whereas Wnt1-Cre; Rosa26^Tomato^ was constitutively active. We found that, within the first week post-surgery, not only a larger number of labelled stem cells reside on the inter-frontal suture when compared to the sagittal, but a higher proportion of them infiltrate the frontal wound in comparison to the parietal. Moreover we provide insights on the neural crest domain at the sagittal suture, which suggest that the position in relation to the surrounding sutures might be a determinant on the efficiency of parietal wound healing.

# Abstract: Postnatal skull development of c-Fos mice and the importance of osteoclasts across ontogeny

**Authors:** Heather White, SE Evans, AS Goswami, AS Tucker

**Affiliations:** Department of Craniofacial Development and Stem Cell Biology, Floor 27 Guy's Tower, King's College London, London Bridge, London, SE1 9RT

The dynamic nature of bone remodelling enables skeletal elements to adapt to their environment, thus playing a crucial role in normal development. Here, we investigated the interaction between abnormal bone remodelling (c-Fos knockout mice) and postnatal skull growth by utilising 3D morphometrics to characterise phenotypic change and link such changes to cellular involvement. MicroCT scans indicated a delayed development of c-Fos mutants compared with WT littermates, illustrated by their domed skull phenotype which lacked tooth eruption and displayed altered morphology of several bones: parietal, nasal and mandible. The two principle components (PCs) contributing to overall shape variation were skull height (37%) and width (31%) with the c-Fos mutants occupying the heightened and wider region of morphospace. Interestingly, this phenotype of the mutants increasingly diverged from WT littermates across postnatal ontogeny. Unexpectedly, microCT reconstructions indicated premature suture fusion (craniosynostosis) in c-Fos mutants. In WT mice at early stages of postnatal development (P4), TRAP-positive osteoclasts lined the bone margins along the patent coronal and sagittal sutures. At later stages of development (P20), osteoclast activity had decreased and was associated with increased bone deposition at the suture locale. Our results suggest an unappreciated role for osteoclasts in maintain suture patency across ontogeny.
